# Leprosy in children under 15 years of age in Brazil: A systematic review of the literature

**DOI:** 10.1371/journal.pntd.0006788

**Published:** 2018-10-02

**Authors:** Michelle Christini Araújo Vieira, Joilda Silva Nery, Enny S. Paixão, Kaio Vinicius Freitas de Andrade, Gerson Oliveira Penna, Maria Glória Teixeira

**Affiliations:** 1 Nursing School, Federal University of the *Vale do São Francisco*, Petrolina, Pernambuco, Brazil; 2 Institute of Collective Health, Federal University of Bahia, UFBA, Salvador, Brazil; 3 Department of Infectious Disease Epidemiology, Faculty of Epidemiology and Population Health, London School of Hygiene & Tropical Medicine, London, United Kingdom; 4 Health Departament, State University of Feira de Santana, Feira de Santana, Bahia, Brazil; 5 Tropical Medicine Center, Brasília University, Brasília, Distrito Federal, Brazil; 6 School of Government of the Oswaldo Cruz Foundation, Brazilian Ministry of Health, Brasília, Distrito Federal, Brazil; Hospital Infantil de Mexico Federico Gomez, UNITED STATES

## Abstract

**Background:**

Leprosy is a chronic infectious disease neglected, caused by *Mycobacterium leprae*, considered a public health problem because may cause permanent physical disabilities and deformities, leading to severe limitations. This review presents an overview of the results of epidemiological studies on leprosy occurrence in childhood in Brazil, aiming to alert health planners and managers to the actual need to institute special control strategies.

**Methodology/Principal findings:**

Data collection consisted of an electronic search for publications in eight databases: *Literatura Latino-Americana e do Caribe em Ciências da Saúde* (LILACS), Scientific Electronic Library Online (SciELO), PuBMed, *Biblioteca Virtual em Saúde* (BVS), SciVerse Scopus (Scopus), CAPES theses database, CAPES journals database and Web of Science of papers published up to 2016. After apply selection criteria, twenty-two papers of studies conducted in four different regions of Brazil and published between 2001 and 2016 were included in the review. The leprosy detection rate ranged from 10.9 to 78.4 per 100,000 inhabitants. Despite affecting both sexes, leprosy was more common in boys and in 10-14-year-olds. Although the authors reported a high cure proportion (82–90%), between 1.7% and 5.5% of the individuals developed a disability resulting from the disease.

**Conclusions/Significance:**

The findings of this review shows that leprosy situation in Brazilian children under 15 years is extremely adverse in that the leprosy detection rate remains high in the majority of studies. The proportion of cases involving disability is also high and reflects the difficulties and the poor effectiveness of actions aimed at controlling the disease. The authors suggest the development of studies in spatial clusters of leprosy, where beyond the routine actions established, are included news strategies of active search and campaigns and actions of educations inside the clusters of this disease. The new agenda needs to involve the precepts of ethical, humane and supportive care, in order to achieve a new level of leprosy control in Brazil.

## Author summary

Leprosy remains as a severe health problem in Brazil and its transmission in children under 15 years of age occurs mainly through intradomiciliary contacts. The number of leprosy cases in this age group is considered an important indicator for the surveillance of this disease. To understand how the epidemiological studies in Brazil have shown the situation of young people affected by leprosy, we performed a systematic review of the literature, searching for published articles about the situation of leprosy in this age group. We reviewed 22 studies published during 2001 to 2016 and concluded that the negative effects of leprosy still remain high in most of studied places in Brazil. This disease was more common in boys, aged between 10 to 14 years old, with a remarkable proportion of disabilities due to leprosy. These disabilities can limit their routine activities and reflect failure in the public medical care. We hope that our review should contribute to arguments in order to improve the control of this disease in children.

## Introduction

Leprosy is a chronic infectious disease caused by *Mycobacterium leprae*. Since the disease may cause permanent physical disabilities and deformities, leading to severe limitations in individual’s ability to perform daily activities, this disease is considered a public health problem worldwide [1, 2]. The incubation period of *M*. *leprae* is very long, in some cases up to ten years, and for this reason the majority of cases only become clinically detectable in adulthood. The occurrence of leprosy in children under 15 years of age suggests early exposure and persistent transmission of the agent [3].

Leprosy control has improved markedly around the world over the past thirty years. During this period the leprosy prevalence fell from 21.1 per 10,000 inhabitants in 1983 to 0.24 per 10,000 inhabitants in 2000. This decline occurred due to the generalized use of multidrug therapy (MDT), in addition to nationwide campaigns and an improvement in the quality of health services directed to leprosy treatment in endemic countries [1]. In 2016, the World Health Organization (WHO) launched a new global strategy entitled “The Global Leprosy Strategy 2016–2020: Accelerating towards a leprosy-free world” [4].

The total number leprosy new cases registered by the WHO in 2016 was 214,783 (2.9 per 100,000 inhabitants), 95% of which are concentrated in only 14 countries of high endemicity where over 1,000 new leprosy cases are notified each year [1]. These countries are geographically situated within the tropics, with India in first place and Brazil coming second in terms of the number of cases detected annually.

In 2017, the total number of leprosy new cases in Brazil was 22,940, of which 1,718 were in children under 15 years of age, corresponding to 7.5% and detection rate of 3.72 cases per 100,000 inhabitants [5]. One of the goals of the Global Leprosy Strategy 2016–2020 is to further reduce global and local leprosy burden, aiming to reduce to zero the number of children with disabilities due to this disease. However, in the last five years in Brazil, the percentage of children <15 years old that has grade 2 disability (G2D) ranged from 2.9% (2013) to 4.1% (2017), with an average proportion of 3.7%, reflecting long delay in diagnosis [5].

One of characteristics of the distribution of leprosy is the occurrence in clusters, and in Brazil the leprosy detection rate in the all population and in the under 15 years of age, varies greatly among regions and cities. A region is considered hyperendemic when the leprosy detection rate in under 15 years of age is above 10 per 100,000 inhabitants [6], in many areas, the values of this indicator reach higher levels those considered hyperendemic [5].

In 2016, the detection rate of new cases in this country reached 12.2 per 100,000 inhabitants, considered “high” according to the reference parameters established by the Ministry of Health [5]. In the northern region, this indicator in the general population was 28.7 per 100,000 inhabitants, and of 8.92 per 100,000 inhabitants under 15 years of age. In the Midwest, 30.01 new cases per 100,000 inhabitants were registered for the general population and 6.42 per 100,000 inhabitants under 15 years of age. In the Northeast of the country, the detection rate in the general population was 19.29 per 100,000 inhabitants, while in those under 15 years of age was 5.78 per 100,000 inhabitants [7, 8].

Based on fact that leprosy in children under 15 years of age is hiperendemic in Brazil and that produce important negative effect on life of the affected children and their families, the research question that guided this systematic review was: Does the epidemiological studies on leprosy in childhood, in Brazil, have warning to the policy makers and health managers that the severity of negative effects of this disease is demanding immediate attention? This review presents an overview of the results of epidemiological studies on leprosy occurrence in children under 15 years, in Brazil, aiming to alert health planners and managers to the need to institute special control strategies for this disease, which is one of the most neglected problems of public health.

## Methods

This study was conducted according to the guidelines established in the Preferred Reporting Items for Systematic Reviews and Meta-Analyses [9], with the study protocol registered in PROSPERO with reference code CRD42016033006PROSPERO.

### Selection criteria and search

The review was conducted between May 17 and August 10, 2016 using the databases listed in Table 1. Equivalent keywords or subject terms were identified using health sciences descriptors (DeCS), a trilingual structured vocabulary created by the Latin American and Caribbean Center on Health Sciences Information (LILACS/BIREME) [10], in the three languages (Portuguese, English and Spanish) included in this review. A simulated search by language was then conducted to verify which terms would achieve the optimal results in each database. The appropriateness of the language used in the search was assured by preserving the three languages as filters in the databases.

**Table 1:** Details of the database search procedure.

Database	Website	Date
CAPES theses database	http://bancodeteses.capes.gov.br/	10/Aug/2016
Biblioteca Virtual em Saúde–(BVS)	http://brasil.bvs.br/	17/May/2016
Literatura Latino-Americana e do Caribe em Ciências da Saúde (LILACS)	http://lilacs.bvsalud.org/	17/May/2016
CAPES Journals	http://www-periodicos-capes-gov-br.ez21.periodicos.capes.gov.br/index.php?option=com_phome	28/July/2016
PubMed	http://www.ncbi.nlm.nih.gov/pubmed	17/May/2016
Scientific Electronic Library Online (SciELO)	http://scielo.org/php/index.php	05/June/2016
SciVerse Scopus (Scopus)	https://www.scopus.com/	07/June/2016
Web of Science	https://apps.webofknowledge.com/UA_GeneralSearch_input.do?product=UA&search_mode=GeneralSearch&SID=3D42qcvkZTMW85yA9LR&preferencesSaved =	06/June/2016

Although the term “children under 15 years of age” was used as a keyword in various studies conducted in Brazil and internationally, no such equivalent was found in the DeCS, with “minors” being the closest term found. Therefore, in addition to the terms *child and adolescent*, the term “*children under 15 years of age*” was also used. Likewise, after a preliminary reading of the international papers, it was found that two forms were used in international papers when referring to “children under 15 years of age”: “under 15 years” and “younger than 15 years”. Therefore, the following search terms were used and restricted to the fields “title”, “abstract” and “keywords”:

*Brazilian databases*: **BVS, CAPES theses database and Scielo**: Hanseníase AND “Epidemiologia” AND “Criança”; Hanseníase AND “Epidemiologia” AND “Adolescente”; “Hanseníase AND “Epidemiologia” AND “Menores de 15 anos”.

*International databases*: **LILACS, CAPES journals, PubMed, Scopus, Web of Science:** "Leprosy" AND "Epidemiology" AND “Child”, "Leprosy" AND "Epidemiology" AND “Adolescent”, "Leprosy" AND "Epidemiology" AND “Under 15 years” OR “Younger than 15 years”, “Lepra” AND “Epidemiología” AND “Niño”, “Lepra” AND “Epidemiología” AND “Adolescente”, “Lepra” AND “Epidemiología” AND “menor de 15 años”.

The protocol defined the following inclusion criteria: complete papers and theses available in the selected databases in English, Portuguese or Spanish. Since there were few scientific papers specifically on leprosy in children under 15 years of age, a decision was made not to restrict the beginning of the search period to any specific year, thereby including all the articles, dissertations and theses found up to the cut-off date of August 2016, in which the type of epidemiological study was described in the Methods section.

The exclusion criteria consisted of papers involving animals; articles published as a short communication or poster; papers in which the methodology used was not described; any other type of documents; and duplicates.

The data extracted from the selected papers were transferred to an Excel 6.0 version spreadsheet using double data entry and evaluated independently by two reviewers (MCAV and KVFA). All disagreements were settled by a third reviewer (MGT).

The relevant data that were collected in the publications selected for the systematic review were: authors, year of publication, journal, study site, study period, study design, population, age group, sex, education, type of housing, leprosy detection rate, epidemiological characterization, operational classification, clinical classification, vaccination with Calmette-Guérin Bacillus (BCG) scar, smear test result, disability degree at diagnosis, disability degree at cure, Hansen's reaction, close contact with leprosy patient, contact examination, 2nd BCG in contacts, detection mode, reason for discharge, complete treatment, relapse and sequelae.

### Evaluation of the quality of studies

A scale was created to evaluate the quality of the articles selected for this systematic review (S1 Table). This scale was based on and adjusted according to the proposals made by Downs and Black [11] and Boas and Neto [12]. Scores were awarded for the selected items on a 17-point scale and analyzed according to the mean score awarded for the quality of the articles.

## Results

**Fig 1:**
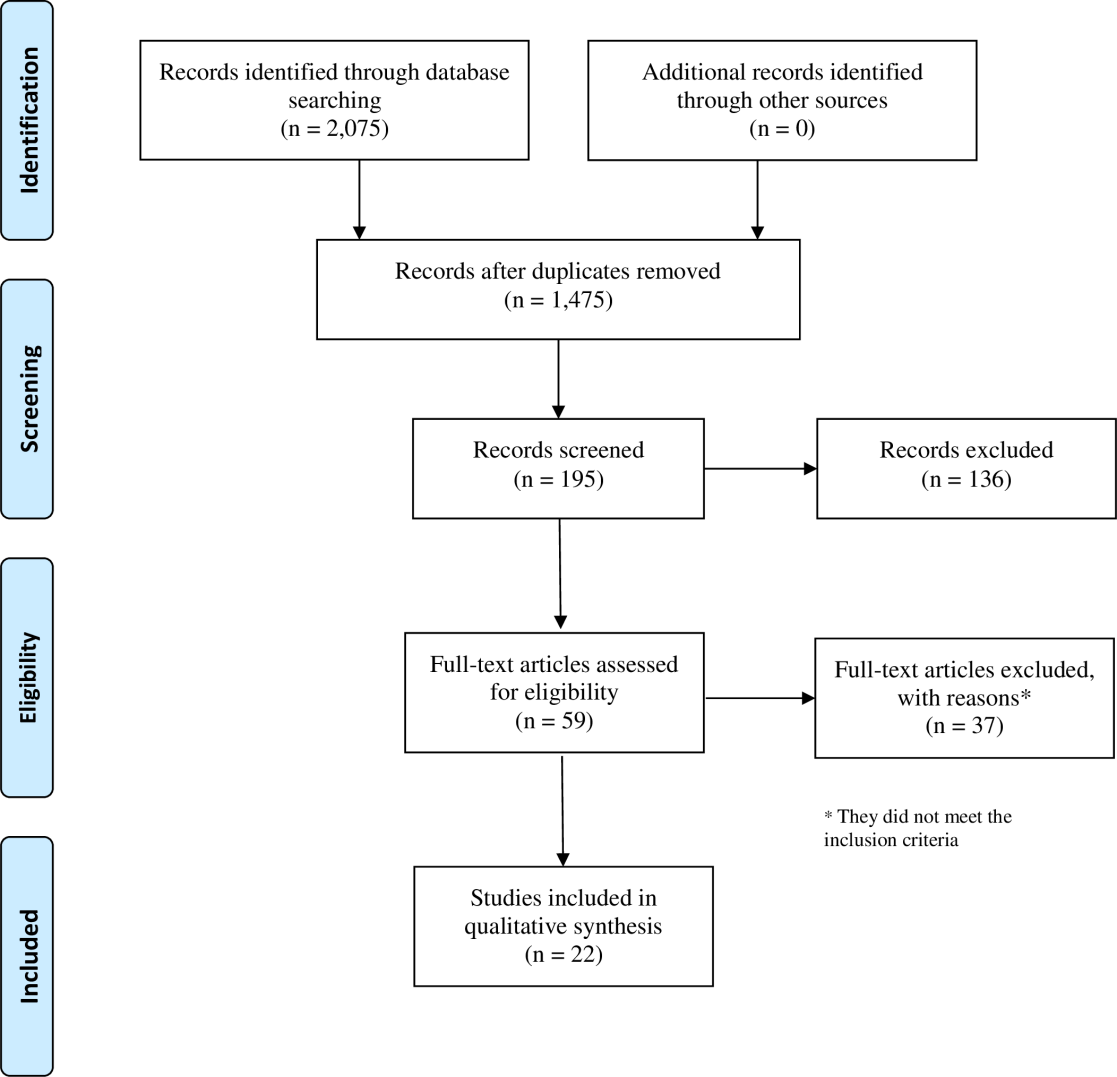
PRISMA flow diagram for the systematic review. Accordingly, the titles and abstracts of 195 publications were evaluated. Fifty-nine of these papers met the inclusion criteria and were then selected to be read in their entirety. After the three reviewers had read these publications, 22 studies (articles, theses and dissertations) were selected for inclusion in this review (Table 2).

A total of 2,075 papers were identified in the databases following a search based on the selected keywords. Subsequently, 1,880 articles were excluded because: they did not meet the study objectives, they dealt with subject matter other than that proposed, duplicates, the papers were not available in their entirety, the age group included in the study was older than that established for this review, or the objective of the study was not included in the keywords, title and/or abstract. Fig 1 consists of a flowchart depicting the selection of the articles in accordance with the PRISMA guidelines.

**Table 2:** Details of the papers selected, including author, year, title, journal and database.

Ref	Author/Year	Title	Journal	Database
**BRAZIL**				
[13]	Levantezi, 2014	**Leprosy in children under fifteen years in Brazil, 2011**	Lepr Rev	CAPES Journals
**Northeast**				
[14]	Alencar et al., 2008	**Hanseníase no município de Fortaleza, CE, Brasil: aspectos epidemiológicos e operacionais em menores de 15 anos (1995–2006)**	Rev Bras Enferm	Web of Science
[15]	Bastos, Raquel Patriota Cota 2010	**Hanseníase: estudo epidemiológico e clínico dos casos ocorridos em menores de 15 anos no estado de Alagoas, no período de 1990–2007**	UFAL thesis	CAPES database of theses
[16]	Luna et al., 2013	**Perfil clínico-epidemiológico da hanseníase em menores de quinze anos no município de Juazeiro-BA**	Rev Bras Promoc Saúde	Biblioteca Virtual em Saúde–(BVS)
[17]	Moreira et al., 2014	**Epidemiological situation of leprosy in Salvador from 2001 to 2009**	An Bras Dermatol	Web of Science
[18]	Oliveira, Carlos Alberto Rodrigues de, 2008	**Perfil Epidemiológico da Hanseníase em Menores de 15 Anos no Município de Teresina**	Dissertation ENSP	Biblioteca Virtual em Saúde–(BVS)
[19]	Santos et al., 2015	**Leprosy and Disability in Children Younger Than 15 Years in an Endemic Area of Northeast Brazil**	The Pediatric Infectious Disease Journal	Biblioteca Virtual em Saúde–(BVS)
[20]	Santos et al., 2016	**Leprosy in children and adolescents under 15 years old in an urban centre in Brazil**	Mem Inst Oswaldo Cruz	SciVerse Scopus (Scopus)
[21]	Souza et al., 2001	**Aplicação de modelo bayesiano empírico na análise espacial da ocorrência de hanseníase**	Rev Saúde Pública	Biblioteca Virtual em Saúde–(BVS)
[22]	Souza et al., 2012	**Epidemiological Profile of Leprosy in the Brazilian state of Piauí between 2003 and 2008**	An Bras Dermatol	Biblioteca Virtual em Saúde–(BVS)
**Southeast**				
[23]	Araújo et al., 2004	**Detecção da hanseníase na faixa etária de 0 a 14 anos em Belo Horizonte no período 1992–1999: implicações para o controle**	Rev Med Minas Gerais	Biblioteca Virtual em Saúde–(BVS)
[24]	Ferreira et al., 2005	**Hanseníase em menores de quinze anos no município de Paracatu, MG (1994 a 2001)**	Rev Bras Epidemiol	Scientific Electronic Library Online (SciELO)
[25]	Ferreira, Maria Aparecida Alves 2012	**Evolução das taxas de detecção de casos de hanseníase em menores de 15 anos no estado de Minas Gerais de 2001 a 2010**	UFMG Thesis	CAPES database of theses
[26]	Flach et al., 2010	**Análise da série histórica do período de 2001 a 2009 dos casos de hanseníase em menores de 15 anos, no Estado do RJ**	Hansen Int	Biblioteca Virtual em Saúde–(BVS)
[3]	Lana et al., 2007	**Hanseníase em menores de 15 anos no Vale do Jequitinhonha, Minas Gerais, Brasil**	Rev Bras Enferm	Web of Science
[27]	Morais et al., 2010	**Avaliação das ações de controle da hanseníase no município de Governador Valadares, Brasil, no período de 2001 a 2006**	Hansen Int	Biblioteca Virtual em Saúde–(BVS)
**Midwest**				
[28]	Freitas, Bruna Hinnah Borges Martins de 2015	**Indicadores e determinantes clínicos e epidemiológicos de hanseníase em menores de quinze anos, Mato Grosso, Brasil**	Dissertation UFMG	CAPES database of theses
**North**				
[29]	Barreto et al., 2012	**High rates of undiagnosed leprosy and subclinical infection amongst school children in the Amazon Region**	Mem Inst Oswaldo Cruz	Web of Science
[30]	Coelho Júnior, José Maria dos Santos, 2010	**Tendência da incidência de hanseníase em menores de 15 anos em Jacundá-Pará e sua relação com a implantação dos serviços de saúde**	Dissertation UFPA	CAPES database of theses
[31]	Franco, Mariane Cordeiro Alves, 2014	**Dinâmica de transmissão da hanseníase em menores de 15 anos em área hiperendêmica na região Norte do Brasil**	Thesis UFPA	CAPES database of theses
[32]	Guimarães, Andréa De Cassia Lima, 2011	**Prevalência de incapacidades sensitivomotoras por doença hansênica em pacientes menores de 15 anos**	Dissertation UFPA	CAPES database of theses
[33]	Imbiriba et al., 2008	**Perfil epidemiológico da hanseníase em menores de quinze anos de idade, Manaus (AM), 1998–2005**	Rev Saúde Pública	Web of Science

The 22 studies selected for inclusion [3, 13–33] were published between 2001 and 2016, and the most productive year was 2010 (04 papers). The predominant database was the BVS from which eight papers were retrieved and included in the review. Geographically, the papers identified came from four regions of the country: the Northeast, Southeast, Midwest and North, as described in Table 2.

All studies included in this systematic review were based on scientific research (n = 22). Most of the studies were linked to universities (n = 20) and only two were from the Brazilian Ministry of Health. The studies were financed by the authors' own resources (n = 14), by the National Scientific and Technological Development—CNPq (n = 06) and Ministry of Health (n = 02).

S2 Table 2 describes the papers in greater detail: author, year of publication, type of study, study population, study period, objective, main findings and score according to the methodology validation scale. The studies were ecological, cross-sectional or case series descriptive. Sample sizes ranged from 24 to 2,455 individuals of both sexes. Most of the studies provided descriptions of the participants according to age (0–4 years, 5–9 years and 10–14 years), sex, detection rate, epidemiological classification, operational classification, clinical classification and degree of disability. Of the 14 publications in which leprosy was presented proportionally by sex, males were more affected in 8. With respect to the distribution of cases according to age group, the greatest number of cases occurred in 10-14-year-olds (n = 10). Only 13 papers provided the clinical classification of the disease and in 6 of these articles the most common was tuberculoid leprosy. Operational classification was described in 16 studies, with the paucibacillary form being predominant in 14. The classification of the epidemiological situation of the studies place was described in 17 articles, with 11 reporting hyperendemic levels in children under 15 years of age, with detection rates that ranged from 10.9 to 78.4 per 100,000 inhabitants. Of these 19 papers, 5 reported prevalence in the states of Alagoas, Minas Gerais, Mato Grosso, Pará and Rio de Janeiro, with the level being highest in Mato Grosso at 25.9 per 100,000 inhabitants in 2014.

The proportion of cases diagnosed with grade 2 disability (n = 13) ranged from 1.7% to 5.5%. However, the percentage of individuals in this group who had not undergone evaluation (n = 5) ranged from 2.4% to 19.20%. Only five articles reported the proportion of grade 2 disabilities following cure (ranging from 0.5% to 11.1%) and only three reported on the proportion of the sample that was not evaluated, citing percentages that ranged from 18% to 61.5%.

Household contact, with the family being the main source of transmission, was reported in three publications. According to the reports, 40% of the children had parents with leprosy, 20–36% a grandparent, 18% an uncle or aunt, and 4% had siblings with the disease.

The examination of contacts was reported in four publications, with 24.2% to 90% of contacts having been examined. The principal form of detection reported (n = 8) was through spontaneous demand at healthcare facilities (13.8% to 79.6%), followed by referral from another healthcare service (12.4% to 75.6%). Other forms of detection such as collective examinations (n = 6) and the examination of contacts (n = 7) were also reported, with percentages ranging from 1% to 6.6% and from 8.9% to 20.4%, respectively.

The presence of a BCG vaccination scar and of a second dose of this vaccine for the contacts was described in only two studies, with results showing that the presence of a scar from the first dose of this vaccine was high; however, revaccination of contacts was very low, between 13.8% and 15.5%.

Five studies reported on acid-fast microscopy results, with this test having been performed in 20.6% to 94.6% of cases and with positivity ranging from 4.3% to 15%. In 4.4% to 79.3% of cases, this test was not performed.

Only two papers described the occurrence of a leprosy reaction. In one of these articles, reaction occurred in 33.4% of patients: immediately following diagnosis in 24.4% and following cure in 9%. The other study simply mentioned that 44.1% of the patients did not develop a leprosy reaction.

Complete treatment involving six doses of multidrug therapy (MDT) was administered in 41.7% to 66.7% of cases, with 12 doses of MDT being administered in 11.8% to 45.8% of cases. In another 2.14% to 21.5% of cases, treatment regimens were longer or unknown. Recurrences were recorded in 3.4% of children. Complete cure ranged from 81.9% to 90% of cases. Few studies emphasized the importance of drawing the attention of health planners and managers to the need to develop special actions for childhood.

## Discussion

This systematic review included 22 articles and showed that the detection rate of leprosy in children under 15 years of age, in Brazil, comes decreasing, but it remains very high in the most of sites investigated reaching levels compatible with hyperendemicity, or at least high or moderate endemicity. It was shown strong association between poverty and leprosy and that its spatial distribution is in clusters. The boys were the most affected by this disease; the proportion of multibacillary cases was high (23.4% to 75%) considering that they were children; the proportion of cases with grade 2 disability varied in the different studies analyzed, from moderate (1.7% to 5.5%) to high at the time of bacteriological cure (18% to 61.5%)and; the proportion of reaction leprosy was also high (33.4% to 55.1%). The coverage of intradomiciliary contacts exams did not reach satisfactory levels, as well as was very low the coverage of the second dose BCG.

These findings clearly indicate that the socio environmental conditions for determination of leprosy occurrence in Brazil are maintained, despite efforts, which have been undertaken to eliminate it, in the last three decades, when there was prevalence reduction near 94%, in total population. These efforts has made possible the continuous reduction in the prevalence and leprosy detection rate in adults and in children in this country as a whole [34], but has not been able to avoid the continued transmission and expansion of this disease that persist in the clusters, particularly in the Midwest, North and Northeast regions [35]. Such situation shows the early contamination of young people, consequent to the failure to detect and treat timely adults bacilliferous cases who maintains the chain of transmission in communities [20]. Luna et al. [16] and Santos et al [20] demonstrate that the distribution of new cases of leprosy differs from area to area, and highlight the need to sensitize managers to perform actual diagnosis by region, with actions being prioritized according to the epidemiological profile. In this perspective, the implementation of health education measures would theoretically bring benefits, mainly in the major cities, where leprosy has been found concentrated in bounded clusters.

The fact that children with leprosy were mostly from families living in great social vulnerability related to low socioeconomic conditions [24, 25, 32] is not a new or unknown fact. It is not by chance, that some of the studies included here reveal that these children have more difficulties in progressing in schooling, that will result in the future in perpetuating poverty, similar to their parents. This is not determined by the physical damages that the disease causes, but by the disease stigma and insufficient social protection. Unfortunately, this situation has been maintained until the present day as more recent studies show. For example, it was found a statistically significant association between greater coverage by the conditional cash transfer program *Bolsa Família* and reduction in the detection rates of leprosy. The authors argue that, in view of its characteristics, this social protection strategy is capable of acting on different social determinants of leprosy such as poverty and economic inequality [36, 37].

As Brazil´s Leprosy Control Programme is integrated in the primary health care while in other countries leprosy interventions are developed in vertical programmes, it is difficult to establish comparisons among endemic countries. Although in Brazil the actions of this Programme are implemented in the whole network of public health services one of difficulties faced is that the majority of new cases reported are known from passive detection by these services, in which the demand is mostly constituted by poor population. There is evidence that when active case search is implemented, unexpectedly higher new case detection rates have been found, even in cities with the highest human development index (HDI), as in São Paulo [38] and Brasília-District Federal [39].

As regards to the higher frequency of boys with leprosy observed in the analyzed studies, further studies need to be conducted to clarify whether boys are indeed at a greater risk of acquiring the disease. If confirmed, a hypothesis to explain this fact could be their greater exposure to social interactions more frequent and intense when compared to girls, increasing the possibility of contact with people with leprosy outside the dwelling [25].

As we know, the basic strategy for the control of leprosy focuses on eliminating the sources of infection using MDT. For this strategy to be effective it has to be adopted timely, i.e. as early as possible to ensure that patients are protected from developing a disability and to reduce the time of transmissibility of the agent. Nevertheless, the proportions of severe disability found in this review are clear proof that diagnosis of the disease is being made late, increasing the risk of nerve damage [26], producing negative repercussion on the daily life of this young. Undoubtedly, physical disabilities negatively affect child’s development, stigmatizing the individual, causing severe psychological repercussions and affecting his/her social life, and it can also reduce a person’s future ability to enter the job market [14, 26, 33]. On the other hand, the high rates of leprosy reaction found highlight the need to increase the availability of specialist services for the prevention of physical disabilities in young people affected by this disease [24]. Not by chance, one of the principal goals of the new Global Leprosy Strategy 2016–2020 [4], is to eliminate grade-2 disabilities in pediatric patients by implementing quality services aimed principally at women and children.

In addition, the predominance of tuberculoid form indicates that immunocompetent individuals also are being affected, highlighting that *Mycobacterium leprae* is circulating intensely [3, 17, 24, 40]. Another finding that gives further strength to this affirmative is the proportion of multibacillary cases which cannot be considered low for the age group under 15 years of age. On the contrary, these cases are concerning, since means that the young people most susceptible to the disease are becoming infected at a very early age, representing yet more one evidence that the process of transmission is intense and continuous [21]. The low proportion the healthy contacts vaccinated, measured by the scar presence of the BCG second dose of the contacts, found in the present review, shows possible failure of surveillance to avoid multibacillary forms.

The main limitation of this systematic review was the low quantity of studies available in the scientific literature, highlighting how much leprosy is a neglected disease and, in particular, when it refers to childhood. In addition, most of these studies were descriptive, using surveillance secondary data that, in general show some inconsistency in relation to the quality and quantity of information. These limitations did not allow the analysis of individual and collective risk factors among other epidemiological aspects.

### Conclusions

In spite of these limitations, the results of this study will be useful, especially for public health, since they will help to alert the decision makers to the need in identifying the care needs of those under 15 years of age affected by the disease due to social stigma and the leprosy physical repercussions in the children.

Although it has not been the object of evaluation of most of the studies, several authors point out that one of the problems of leprosy control is the fragility of surveillance, since the action of health services is predominantly individualized, with a low proportion of search active contacts. The detection of new cases depends on the spontaneous demand of the individual, who mostly seek the health service in advanced stage of the disease, which increases the risk of permanent damage. [3, 5, 19, 23].

The presence of the contacts and the communities in the greatest risk of disease, should be found of the pillars of collective actions, continues being one of the main force to overcome those in the Brazilian leprosy control program. Besides, the treatment of leprosy patients still not meet the target established (98%) by Brazilian Ministry of Health and WHO [25].

In view of this adverse scenario, we suggested the development of studies in spatial clusters of leprosy, where beyond of the control and surveillance actions established by the Brazilian Ministry of Health, are included news strategies of active search adding social contacts, campaigns and actions of educations inside these areas aiming early diagnoses and treatment. If many new cases in children will be discovered in these communities, it is understood that the search must be expanding to others neighboring area to uncover the primary sources of infection to prevent new cases in childhood.

For this, it is necessary a debate and initiatives needs to be carried out to establish an agenda aimed at strengthening the active surveillance of this disease. This agenda needs to involve the precepts of ethical, humane and supportive care in order to achieve a new level of leprosy control in Brazil.

## Supporting information

S1 Table Methodological validation scale for the articles. (DOCX)

S2 Table Articles selected according to the author, year, type of study, population, study period, objective, principal findings and score according to the methodological validation scale. (DOCX)
