# Supplementary material for: Leprosy in children under 15 years of age in Brazil: A systematic review of the literature
Source: PLoS Negl Trop Dis. 2018 Oct 2;12(10):e0006788. doi: 10.1371/journal.pntd.0006788 (PMC6168122; doi:10.1371/journal.pntd.0006788)
Supplement: S1 Table — (DOCX) [file pntd.0006788.s001.docx]

**S1 Table. Methodological validation scale for the articles.**

| **Methodological validation scale of the articles** | **Yes**  **(1 point)** | **No**  **(0 point)** | **N/A** |
| --- | --- | --- | --- |
| 1. Appropriate use of the scientific literature? |  |  |  |
| 1. Objectives and/or hypotheses are clearly defined? |  |  |  |
| **Methodology** |  |  |  |
| 1. Is the type of study described in the Introduction or Methods sections and developed correctly? |  |  |  |
| 1. Are the methods present and presented consistently?   Has the study site and period been included? Have the selection criteria (inclusion and exclusion) for the participants been described? Have the data source, data collection and data analysis been described? |  |  |  |
| 1. Have the outcomes been presented correctly? |  |  |  |
| 1. Is the sample in consonance with the objectives? |  |  |  |
| 1. Was the sample size calculated? |  |  |  |
| 1. Were the analysis and statistical tests adequately used? |  |  |  |
| 1. Has the existence or absence of conflicts of interest been stipulated? |  |  |  |
| 1. Has an evaluation of validity and accuracy been included? |  |  |  |
| 1. Have the study limitations been listed? |  |  |  |
| 1. Is the study adequate for understanding the epidemiological status of leprosy in children under 15 years of age? |  |  |  |
| 1. Have measures of relative frequency been presented and described? |  |  |  |
| **Results** |  |  |  |
| 1. Have the results been adequately presented? |  |  |  |
| 1. Do the results meet the objectives? |  |  |  |
| **Conclusion** |  |  |  |
| 1. Are the Discussion and Conclusions in line with the principal findings of the study and do these sections addressdata in the literature? |  |  |  |
| 1. Is the paper clearly written? |  |  |  |
| **TOTAL** |  |  |  |

N/A: Not applicable.
