# Supplementary material for: Leprosy in children under 15 years of age in Brazil: A systematic review of the literature
Source: PLoS Negl Trop Dis. 2018 Oct 2;12(10):e0006788. doi: 10.1371/journal.pntd.0006788 (PMC6168122; doi:10.1371/journal.pntd.0006788)
Supplement: S2 Table — (DOCX) [file pntd.0006788.s002.docx]

**S2 Table. Articles selected according to the author, year, type of study, population, study period, objective, principal findings and score according to the methodological validation scale.**

|  | **Author/**  **Year** | **Type of Study** | **Pop.** | **Study Period** | **Objective** | **Principal Findings** | ***** |
| --- | --- | --- | --- | --- | --- | --- | --- |
| 01 | Alencar et al., 2008 | Cross-sectional | 451 | 1995-2006 | To analyze the epidemiological status of leprosy in children under 15 years of age in the city of Fortaleza between 1995 and 2007. | “The epidemiological indicators in this population show hyperendemicity. Operationally, it was found that the majority of patients seen continued to be concentrated at some referral units, despite some advances. Late diagnosis, a high degree of disability at diagnosis and a poor degree of evaluation of registered contacts reveals the fragility of the control actions. The possibility of diagnostic error is emphasized in view of the characteristics of the infection in this population. The occurrence of cases of leprosy in this population represents an epidemiological indicator of great relevance and its analysis adds to the debate on operational issues in the healthcare network”. | 12 |
| 02 | Araújo et al., 2004 | Descriptive | 84 | 1992-1999 | To describe the occurrence of leprosy in children under 15 years of age in the city of Belo Horizonte between 1992 and 1999 through the use of certain epidemiological and operational indicators. | “A total of 1,573 notifications made during this period were evaluated, 84 (5.3%) in individuals under 15 years of age. In Belo Horizonte, 39% of new cases of leprosy occurred in individuals of 15 to 45 years of age. The detection rates were considered high (0.35 and 0.34/10,000 inhabitants) for 1992 and 1993 and moderate for the remainder of the study period (0.09 to 0.14/10,000 inhabitants). The reduction in the detection rates also occurred in the group of individuals over 15 years of age. Examination of household contacts was responsible for 47.6% of the new cases diagnosed in children under 15 years of age. The multibacillary clinical forms - Virchow and dimorphic - were found in 75% of the cases occurring in children under 15 years of age, with an absolute predominance of the dimorphic form in 72.6% of these. The multibacillary forms were also predominant in individuals over 15 years of age, comprising 84.1% of all cases. Overall, 85.7% of cases in children under 15 years of age and 68% of the group over 15 years of age had grade 0 disability (patients with no physical disability). Patients with grade 2 disability comprised 8.3% of the group of children under 15 years of age and 11.2% of those over that age, with the latter being considered high by the Ministry of Health”. | 12 |
| 03 | Barreto et al., 2012 | Cross-sectional | 51 | 2009-2011 | To determine the prevalence of subclinical infection (defined in this study as seropositivity for anti-PGL-I IgM with no clinical signs or symptoms of leprosy) and the prevalence of undiagnosed leprosy amongst school childrenfrom selected municipalities in Pará. | “The results of this study suggest that there is a high rate of undiagnosed leprosy and subclinical infection amongst children in the Amazon Region. The advantages of school surveys in hyperendemic areas include identifying leprosy patients at an early stage when they show no physical disabilities, preventing the spread of the infection in the community and breaking the chain of transmission”. | 13 |
| 04 | Bastos, 2010 | Cross-sectional  Descriptive | 475 | 1997-2007 | To describe the epidemiological and clinical profile of the occurrence of leprosy in children under 15 years of age in the state of Alagoas based on the cases notified to the Alagoas State Health Department between 1990 and 2007. | “The findings showed that 8.9% of the new cases of leprosy detected in Alagoas between 1990 and 2007 were in children under 15 years of age. The epidemiological indicators show the high endemicity of the state, with hyperendemicity in some municipalities. Operationally, it was found that the detection of new cases was predominantly from passive demand and that the examination of household contacts was performed routinely (58.4%). The degree of physical disability at diagnosis was zero in the majority of cases (69.60%), with grade 2 physical disabilities in 3.8% of cases in this age group. The occurrence of cases of leprosy in this age group represents an epidemiological indicator of the utmost relevance, suggesting the need to reevaluate currently applied actions for control of the disease”. | 12 |
| 05 | Coelho Júnior, 2010 | Descriptive and ecological | 210 | 1999-2008 | To investigate and analyzeleprosy incidence in children under 15 years of age in the municipality of Jacundá in a historical series and its association with the implantation of health surveillance services. | “In the years following implementation of health services, an increase was found in the detection rate and the municipality remained hyperendemic in the final nine years of the series (>1.0 case/10,000 inhabitants). However, in the final three years, the incidence decreased, with evidence that the implantation of new services contributed to the new scenario. Most of the patients were in the 10-14-year-age group (64.76%), were male (50.5%) and came from an urban area of the municipality (84.3%). The dimorphic form predominated at the time of diagnosis (42.4%). According to the operational classification, the paucibacillary form was predominant (52.9%), and was attributed to the improvement in diagnosis at early stages. In conclusion, based on the study indicator, there was a trend towards a reductionin endemic levels in the municipality”. | 12 |
| 06 | Ferreira et al., 2005 | Descriptive | 45 | 1994-2001 | To evaluate the clinical and epidemiological characteristics of the patients under 15 years of age registered at the leprosy service in Paracatu, Minas Gerais between 1994 and 2001. | “Overall, 53% of the diagnoses were in boys and 75% of cases were in children between 10 and 14 years of age. At school, there was a discrepancy regarding the appropriate grade for ageand 84% of the clients lived in an urban region. All those registered (100%) were new cases, with 56.0% having the multibacillary form, the dimorphic clinical form, and 44% having paucibacillary forms such as indeterminate leprosy (30.8%) and tuberculoid leprosy (13.2%). All were treated using the standard treatment regimen and the cure rate was 100%. Reactions occurred in 24% of patients at diagnosis and during treatment, while 9% developed reactions following the end of treatment and cure. Overall, 22% of patients had some degree of disability at diagnosis and 13% had some degree of disability following treatment and cure, a rate that was considered high. It was found that 62% of the patients had household contact with individuals with leprosy, with two BCG vaccination scars being found in only 15.5% of these contacts”. | 11 |
| 07 | Ferreira, 2012 | Ecological | 1,378 | 2001-2010 | To analyze the changes in leprosy detection rates in children under 15 years of age in the state of Minas Gerais from 2001 to 2010 and to investigate the influence of socioeconomic variables in the municipalities. | “The leprosy detection rates in children under 15 years of age in the state of Minas Gerais underwent a significant decline from 2004 onwards (p<0.001), although the detection rates remained at moderate to high levels and a trend was also seen in the spatial distribution. In the simple Poisson model, the variables found to be associated with changes in the detection of cases of leprosy in all the years evaluated were the human development index, per capita GDP and themean number of years of schooling of the heads of households, (with a negative coefficient), and the percentage of illiteracy in mothers or mothers-in-law (with a positive coefficient)”. | 12 |
| 08 | Flach et al., 2010 | Retrospective | 1.447 | 2001-2009 | To describe the occurrence of cases of leprosy in the population under 15 years of age in the state of Rio de Janeiro in the preceding nine years using a historical series from 2001 to 2009 according to data provided by the Notifiable Diseases Database (SINAN/NET/SESDEC-RJ). | “The detection rate in this age group went from a level classified as “very high” (up to 2004) to “high” at the end of the study period, showing a trend towards a slow and progressive reduction in endemic levels. The proportion of pediatric cases in relation to the total number of cases remained at 6.6% between 2005 and 2007, with a slight reduction in 2008 and 2009. Paucibacillary forms were predominant in this age group throughout the study period. Evaluation of the control activities that led to a reduction in the risk of nerve damage such as the degree of disability at diagnosis and at cure have shown results considered moderate. The evaluation of children under 15 years of age at diagnosis shows results below those found in relation to the total number of new cases in the state; evaluation at cure was precarious up to 2007, with the best indexes being reached in 2008 and 2009, coming close to the values recommended by the Ministry of Health. The indicators of the study group in general followed the trend towards a decrease seen in the indicators of the total number of cases of leprosy in the state of Rio de Janeiro, with the improvement in the indexes reflecting the success of the strategies used to control the endemic situation and the attention given to preventing disabilities in patients”. | 12 |
| 09 | Franco, 2014 | Ecological and longitudinal | 36 | 2003-2013 | To describe the dynamics of the transmission of leprosy in children under 15 years of age in a hyperendemic municipality in the north of Brazil, taking factors of the risk and territoriality for the disease into consideration. | “Overall, 226 cases of leprosy were notified for all age groups, with 15.92% (36 cases) being in children under 15 years of age, with a decrease in the detection rate over the decade evaluated. In the state of Pará, there was a slight decrease in notifications. In the municipality of Igarapé Açu, there was an expressive increase in the number of new cases in 2005, 2009 and 2011 as a result of campaigns in the schools to diagnose the disease. Adolescents constituted the predominant age group irrespective of sex, confirming the long incubation period of the disease. The education and socioeconomic levels of the families were very low. The predominant dermatological alteration in the group consisted of a single lesion located on the lower limbs.The paucibacillary form was more prevalent, with the identification of two cases of multibacillary leprosy. The rates of treatment dropout and recurrences were relevant, as was the disability index, which signals late diagnosis. With respect to the risk factors for the disease, consanguinity and household contact were shown to be significantly relevant, with no differences between them or between the different degrees of family relationship. The time of contact was important, with a mean of 8.6 years, and among cases of minors who had household contact with an index case the rate of a second BCG vaccination was low. When geocoded annually according to the area covered by the community health agent and associated with the total numbers of paucibacillary and multibacillary forms, a direct correlation was found between the cases of leprosy in children under 15 years of age and multibacillary cases, confirmed by the positive Moran’s I (0.71) and statistically significant p-value (0.019). Geostatistics confirmed that according to the classic epidemiological indicators, cases of leprosy in children under 15 years of age are closely associated with multibacillary cases”. | 12 |
| 10 | Freitas, 2015 | Cross-sectional  Descriptive | 2,455 | 2001-2013 | To analyze the clinical and epidemiological indicators and determinants of leprosy in children under 15 years of age registered at the Notifiable Diseases Database(SINAN/MT, Brazil). | “The first study indicated a mean incidence of 22.7 cases per 100,000 inhabitants under 15 years of age. The majority were boys (51.6%; n=1,268) and children between 10 and 14 years of age (65%; n=1,595). Of the clinical characteristics, 67.3% (n=1,652) were classified as paucibacillary and 45.8% (n=1,125) had a single lesion. Regarding the mode of detection, 46.2% (n=1,133) of entries resulted from spontaneous demand. Regarding whether acid-fast microscopy was performed at diagnosis and whether a leprosy reaction was present, it was found that in 60.6% and 52.1% of cases, respectively, the information was either unknown and/or not registered. In the second study, a trend towards a decrease in the overall incidence rate was found, with a mean annual rate of -5.5% (95%CI: -7.5 - -3.5). There was also a trend towards growth, with an increase of 6.7% (95%CI: 2.7 - 10.8) in the proportion of multibacillary cases, of 9.4% (95%CI: 4.4 - 14.7) in the number of cases diagnosed with the dimorphic clinical form and of 14.0% (95%CI: 7.9 - 20.4) in the number of cases with grade 2 physical disability at the time of diagnosis. There was a growth in the trend of the mean proportion of contacts examined, with an increase of 4.1% (95%CI: 1.2 - 7.1), whereas the mean proportion of cure in the period was considered precarious (39.7%) with a trend towards stagnation. The final study found that 73.9% (n=317) of the new cases were notified in municipalities considered priority. A difference was found between the municipalities with respect to the proportion of cases registered, with a higher proportion in the municipalities classified as priority for: age 5-9 years (χ^2^=4.09; p=0.043), ethnicity white (χ^2^=7.01; p=0.008) and the tuberculoid clinical form (χ^2^=3.89; p=0.048) and a greater proportion in the non-priority municipalities with respect to non-urban regions (χ^2^=24.23; p<0.001), 2-5 lesions (χ^2^=5.93; p=0.014) and spontaneous demand (χ^2^=6.16; p=0.013). Of the 141 state municipalities evaluated according to spatial distribution, 58.1% (n=82) had a mean incidence coefficient rated as high, very high or hyperendemic and 34.1% of these (n=28) belonged to the group considered priority”. | 13 |
| 11 | Guimarães, 2011 | Descriptive | 24 | 2001-2005 | To identify the presence of sensory/motor impairment resulting from leprosy at diagnosis and up to 10 years following cure, in children under 15 years of age receiving care at specialized leprosy referral units. | “The findings of this study describe the profile of the study sample as being principally female, single, with the majority coming from the state capital city of Belem or its greater metropolitan area, with some elementary school education, and being employed in the informal job market, with a family income of one minimum salary. The clinical characteristics of the disease showed a prevalence of the dimorphic type and zero disabilities. The prevalence of sensory/motor impairment was low in the study group. This fact may be associated with an improvement in leprosy surveillance and control by the healthcare services as well as the effectiveness of treatment”. | 10 |
| 12 | Imbiriba et al., 2008 | Descriptive, retrospective | 474 | 1998-2005 | To analyze the epidemiological status of leprosy in children from an urban region. | “The cases of leprosy in children under 15 years of age corresponded to 10.4% of all cases detected in the period. The detection rate in this age group remained at hyperendemic level between 1998 and 2003, decreasing from 2004 onwards but remaining at very high endemic levels. The most common clinical form was tuberculoid, followed by dimorphic. The paucibacillary forms corresponded to 70.7% of the cases and at the time of diagnosis the degree of disability was evaluated in 94.7% of the patients, with 2.9% presenting with physical disabilities. The majority of cases (99.4%) were treated with the multidrug therapy recommended by the World Health Organization”. | 12 |
| 13 | Lana et al., 2007 | Ecological  Cross-sectional | 114 | 1998-2006 | To analyze the epidemiological status of leprosy in children under 15 years of age in the Vale do Jequitinhonha between 1998 and 2005, with a view to increasing available knowledge on the behavior of the endemics in the region and to design more effective actions for the control and elimination of the disease as a public health issue. | “The results show that the endemic status of the disease has been maintained in this region, indicating the passivity of the healthcare services and the need to implement specific measures of prevention and control for this age group aimed at reducing transmission of the disease and the resulting damage to the lives of affected individuals, particularly when affected in childhood”. | 12 |
| 14 | Levantezi, 2014 | Descriptive | 2,420 | 2011 | To describe the occurrence of leprosy in minors under 15 years of age reﬂecting the burden of disease in the same population in Brazil in 2011. | “The study’s results showed that 2,420 minors with leprosy in 2011 were distributed in 5,565 cities. A total of 692 cities registered the occurrence of 1–10 cases of the disease, with a total of 1,489 cases (61%); 35 cities reported 15 to 25 cases, a total of 544 cases (22·5%); and eight cities notiﬁed 25 cases or more, totaling 87 cases (16%), therefore, around one-third of leprosy cases in children under 15 years of age in Brazil in 2011 are concentrated in 43 Brazilian cities”. | 12 |
| 15 | Luna et al., 2013 | Descriptive | 145 | 2001-2010 | To describe the epidemiological and clinical profile of the occurrence of new cases of leprosy in children under 15 years of age notified to the State Health Department, Juazeiro, Bahia, Brazil between 2001 and 2010. | “Results show that 145 new cases of leprosy (7.94%) occurred in children under 15 years of age. Detection rates were high for this age group, with a predominance of girls (n=81; 55.86%) and of children in the 10-14-year-age group (n=85; 58.62%). The paucibacillary forms (n=107; 74.48%) of the disease were more predominant than the multibacillary forms (n=37; 25.52%), with the tuberculoid clinical form being the most prevalent (n=80; 55.17%). Eighteen of the patients (12.41%)were found to have disabilities at diagnosis and 15 (10.34%) at the evaluation following cure. A large proportion of cases (n=58; 40.07%) were either not evaluated or the results of evaluation were unknown”. | 12 |
| 16 | Morais et al., 2010 | Descriptive  Cross-sectional | 218 | 2001- 2006 | To evaluate the epidemiological and operational status of leprosy control in the municipality of Governador Valadares, Minas Gerais, Brazil between 2001 and 2006. | “The high detection rates overall and for children under 15 years of age found in the municipality of Governador Valadares between 2001 and 2006 continue to classify the municipality as hyperendemic for leprosy. Despite the predominance of young adults, the number of individuals under 15 years of age diagnosed with leprosy is high, highlighting the need for preventive actions in this age group. There is a trend towards an improvement in relation to the type of outcome, with an increase in cases of cure and a reduction in treatment dropout from 2005 onwards. An improvement has been seen in control actions in Governador Valadares over the years; however, there remains a major challenge to be faced in the control of leprosy in the municipality”. | 12 |
| 17 | Moreira et al., 2014 | Cross-sectional  Retrospective | 290 | 2001-2009 | To analyze the epidemiological situation of leprosy in Salvadorbetween 2001 and 2009. | “Over these nine years, 3,226 cases were reported, with a predominance of females (51.5%), and of clinical multibacillary forms in the general population (51.7%); however, when those under 15 years of age were analyzed separately, paucibacillary forms (tuberculoid + indeterminate) prevailed. The tuberculoid form was the most diagnosed type of presentation. The annual detection rate in Salvador remained at a very high level of endemicity during the study period,while for those under 15 years of age it ranged between high and very high. Grade 2 disabilities, both at the time of diagnosis and at cure, varied between low and moderate”. | 13 |
| 18 | Oliveira, 2008 | Descriptive | 1,021 | 1997-2006 | To analyze the epidemiological profile of leprosy in children under 15 years of age in the municipality of Teresina between 1997 and 2006. | “Findings show that young people comprised over 10% of cases throughout the entire study period, with no defined transmission patterns between boys and girls. Young people of 10 to 14 years of age were those most affected by the disease; however, there were notifications of the disease in children under five years of age. Despite a reduction in the detection rate in young people, the municipality remains hyperendemic, with a trend towards an increase in the number of multibacillary cases. Another relevant finding was the concentration of cases in the old neighborhoods of this capital city, particularly in the northern region of the city”. | 12 |
| 19 | Santos et al., 2015 | Retrospective | 266 | 2001-2012 | To describe the incidence of leprosy and the prevalence and degree of disability in children under 15 years of age at the time of first presentation to government health services in Aracaju, Sergipe, Northeastern Brazil. | “The mean detection rate was 16.5/100,000 children under 15 years of age per year over the study period. Physical disability was associated with the presence of nerve damage and multibacillary leprosy”. | 13 |
| 20 | Santos et al., 2016 | Ecological | 145 | 2007-2011 | To describe the intra-urban distribution of cases of leprosy in residents under 15 years of age in Salvador, Bahia, Brazil and to identify the environment in which *M. leprae* is being transmitted in this age group. | “Between 2007 and 2011, 145 cases of leprosy were reported in the target population living in Salvador, corresponding to detection rates of 6.21, 6.14, 5.58, 5.41 and 6.88/100,000 inhabitants, respectively. The spatial distribution of the disease was in clusters. Of the 157 neighborhoods of Salvador, 44 (28.6%) had notified cases of leprosy and in 22 (50%) of these more than 10 cases were detected per 100,000 inhabitants. The infectious forms were reported in 40% of cases. Over 90% of cases had been living in Salvador for more than five years. Overall, 52.6% reported having had contact with another infected individual within the household and 25% in their social circle. In Salvador, *M. leprae* transmission is established. The situation constitutes a major concern, since transmission is intense at an early age, indicating that this endemic disease is expanding and contacts extend beyond individual households”. | 13 |
| 21 | Souza et al., 2001 | Bayesian Model | 799 | 1993-1997 | To supply an overview of the territorial distribution of the disease using crude and adjusted detection rates, analyzing the differences between these distributions and identifying areas of possible under-registration of cases or of probably high transmission (risk) based on an ecological approach, and to shed light on the actual endemic process. | “The mean annual occurrence of new cases in children under 15 years of age was 17.3% (28.3% of which consisted of multibacillary forms), indicating a process of intense transmission of the disease. Analysis of the spatial distribution of leprosy indicated three areas in which the neighborhoods with high detection rates were concentrated and where life conditions were poor”. | 13 |
| 22 | Souza et al., 2012 | Retrospective  Descriptive | 1,040 | 2003-2008 | To evaluate the clinical and epidemiological profile of leprosy patients between 2003 and 2008 in the state of Piauí; to analyze detection and prevalence rates in the general population and in the population of children under 15 years of age; and to evaluate the predominant clinical forms. | “Of the 12,238 cases of leprosy reported in this period, 85% represented new cases. The mean overall annual detection rate was 54 cases/100,000 inhabitants. The rate for children under 15 years of age was 15.3 cases/100,000 inhabitants. Overall, 52.18% of the patients were male; 64.66% were between 20 and 59 years of age; and 53.53% had the paucibacillary form of leprosy. Nevertheless, in 88.82% of cases of the paucibacillary form of the disease, more than five lesions were present, while in 10.55% of cases of the multibacillary form of the disease, no lesions were present. Over 20% of patients had some degree of disability”. | 11 |

*Score according to the methodological validation scale.
